# A Practical Nomogram and Risk Stratification System Predicting Cancer-Specific Survival for Hepatocellular Carcinoma Patients With Severe Liver Fibrosis

**DOI:** 10.3389/fsurg.2022.920589

**Published:** 2022-06-16

**Authors:** Dashuai Yang, Yang Su, Fangrui Zhao, Chen Chen, Kailiang Zhao, Xiangyun Xiong, Youming Ding

**Affiliations:** ^1^ Department of Hepatobiliary Surgery, Renmin Hospital of Wuhan University, Wuhan, China; ^2^ Department of Oncology, Renmin Hospital of Wuhan University, Wuhan, China

**Keywords:** severe liver fibrosis, nomogram, cancer-specific survival, risk stratification system, hepatocellular carcinoma

## Abstract

**Objective:**

Hepatocellular carcinoma (HCC) is the second leading cause of cancer-related deaths worldwide. This study aims to construct a novel practical nomogram and risk stratification system to predict cancer-specific survival (CSS) in HCC patients with severe liver fibrosis.

**Methods:**

Data on 1,878 HCC patients with severe liver fibrosis in the period 1975 to 2017 were extracted from the Surveillance, Epidemiology, and End Results database (SEER). Patients were block-randomized (1,316 training cohort, 562 validation cohort) by setting random seed. Univariate and multivariate COX regression analyses were employed to select variables for the nomogram. The consistency index (C-index), the area under time-dependent receiver operating characteristic curve (time-dependent AUC), and calibration curves were used to evaluate the performance of the nomogram. Decision curve analysis (DCA), the C-index, the net reclassification index (NRI), and integrated discrimination improvement (IDI) were used to compare the nomogram with the AJCC tumor staging system. We also compared the risk stratification of the nomogram with the American Joint Committee on Cancer (AJCC) staging system.

**Results:**

Seven variables were selected to establish the nomogram. The C-index (training cohort: 0.781, 95%CI: 0.767–0.793; validation cohort: 0.793, 95%CI = 95%CI: 0.779–0.798) and the time-dependent AUCs (the training cohort: the values of 1-, 3-, and 5 years were 0.845, 0.835, and 0.842, respectively; the validation cohort: the values of 1-, 3-, and 5 years were 0.861, 0.870, and 0.876, respectively) showed satisfactory discrimination. The calibration plots also revealed that the nomogram was consistent with the actual observations. NRI (training cohort: 1-, 2-, and 3-year CSS: 0.42, 0.61, and 0.67; validation cohort: 1-, 2-, and 3-year CSS: 0.26, 0.52, and 0.72) and IDI (training cohort: 1-, 3-, and 5-year CSS:0.16, 0.20, and 0.22; validation cohort: 1-, 3-, and 5-year CSS: 0.17, 0.26, and 0.30) indicated that the established nomogram significantly outperformed the AJCC staging system (*P* < 0.001). Moreover, DCA also showed that the nomogram was more practical and had better recognition.

**Conclusion:**

A nomogram for predicting CSS for HCC patients with severe liver fibrosis was established and validated, which provided a new system of risk stratification as a practical tool for individualized treatment and management.

## Introduction

HCC is the second leading cause of cancer-related mortality globally and the fifth most severe malignancy (1, 2). The five-year survival rate for patients with HCC is low due to therapeutic restriction (3). The risk factors vary depending on the distribution of the region. For example, chronic hepatitis B virus infection is the primary factor in Asia, while chronic hepatitis C virus infection, alcoholic liver disease, and nonalcoholic fatty liver disease are the prominent risk factors in Europe and America (4–7). A total of 80%–90% HCC patients have biopsy evidence of liver fibrosis (8). Liver fibrosis, a chronic liver injury repair process, is characterized by the activation of hepatic stellate cells into myofibroblasts and the production of large amounts of extracellular matrix, leading to a gradual destruction of the normal structure and physiological function of liver tissue, with scar tissue replacing liver parenchyma and ultimately death, which may eventually lead to cirrhosis, liver failure, or liver cancer (9). Severe liver fibrosis is an irreversible biological process for which no drugs have been proven to be effective (10, 11). Moreover, the management of HCC patients with severe liver fibrosis is extremely controversial in nature (12).

The American Joint Committee on Cancer (AJCC) tumor-node-metastasis (TNM) system is the most commonly used method to evaluate the prognosis of patients with HCC (13). However, the TNM system has some limitations such as low accuracy, ignoring of other factors (age, sex, etc.), and poor performance in predicting individual survival risk (14). As a result, a new and personalized prediction model is needed to evaluate the prognosis of HCC patients.

Recently, clinical models related to nomograms have been widely applied for the survival prediction of tumor patients through a comprehensive analysis of neoplasm-related risk factors (15, 16). Moreover, nomograms can effectively predict tumor prognosis and promote personalized medicine. However, there are no prognostic models for HCC patients with severe fibrosis (Ishak 5–6; Advanced/severe fibrosis; METAVIR F4; Batt-Ludwig 4; Cirrhosis). Therefore, it is necessary to establish a practical, reliable, and specific prediction model to predict CSS for HCC patients with severe liver fibrosis.

## Methods

### Data Collection

Clinically relevant data were extracted from the SEER database between 1975 and 2017 via SEER*Stat 8.3.9.2 software. The SEER database was made publicly accessible and private data for all patients were removed from the database, which indicated that institutional review board approval and informed consent were not required.

### Collation of Data

The inclusion criteria were as follows: (a) HCC patients with severe liver fibrosis (Ishak 5–6; Advanced/severe fibrosis; METAVIR F4; Batt-Ludwig 4; Cirrhosis); (b) complete treatment information. The exclusion criteria were as follows: (a) unknown liver fibrosis score or mild liver fibrosis; (b) metastatic liver cancer; (c) imperfect treatment information; (d) unknown tumor stage; (e) unknown tumor pathological grade; (f) unknown household income; (g) other tumor death and unknown cause of death. Finally, eleven variables were included from the SEER database: age (at diagnosis), ethnicity, gender, tumor number, pathological grade, tumor size, extension, tumor stage (AJCC stage), type of surgery, AFP, and insurance. In addition, the seventh edition of the AJCC-TNM staging was used for the analysis.

### Establishment of the nomogram

All patients were randomly divided into a training cohort and a validation cohort at a ratio of 7:3. The training cohort was used to create a nomogram, while the validation cohort was used for validation. Univariate and multivariate COX regression analyses were employed to obtain significant factors that significantly affected CSS and further construct the nomogram (Supplementary Table).

### Validation of the Nomogram Model

C-index and ROC curves reflected the predictive capability of the nomogram. The value was above 0.5, indicating predictive performance, which could be divided into low precision (0.5–0.7), medium precision (0.71–0.9), and high precision (>0.9). 1-, 3-, and 5- year calibration curves were plotted to evaluate calibrating ability, and the 45-degree line was used as the actual outcome of the primary model.

### Comparison of the Risk Stratification Associated with the Nomogram and AJCC

Based on the nomogram, a novel risk stratification system was developed, which could divide patients into low-, middle-, and high-risk groups (the best cut-off value for the total score was selected by using X-tile). The net reclassification index (NRI), C-index, IDI, and decision curve analysis (DCA) were adopted to compare the risk stratification of the nomogram model with that of the AJCC stage system. The NRI, C-index, and integrated discrimination improvement (IDI) were applied to assess the improvement in risk prediction and determine the effectiveness of the new model. DCA was performed to evaluate the net benefit of various models. Kaplan–Meier curves were used to compare the risk stratification of the nomogram with that of AJCC staging criteria.

### Data Analysis

Univariate and multivariate COX regression analyses, C-index, calibration curves, ROC curves, and DCA curves were generated using R version 4.1.2 and related software packages. The optimal cut-off point for risk stratification was selected utilizing X-tile (version 3.6.1). Statistical differences of distribution between the training and the validation cohorts were analyzed by the Chi-square test. All *p*-values resulted from two-side statistical testing, while a *p*-value less than 0.05 was considered statistically significant.

## Results

### Patient Characteristics

**Figure 1:**
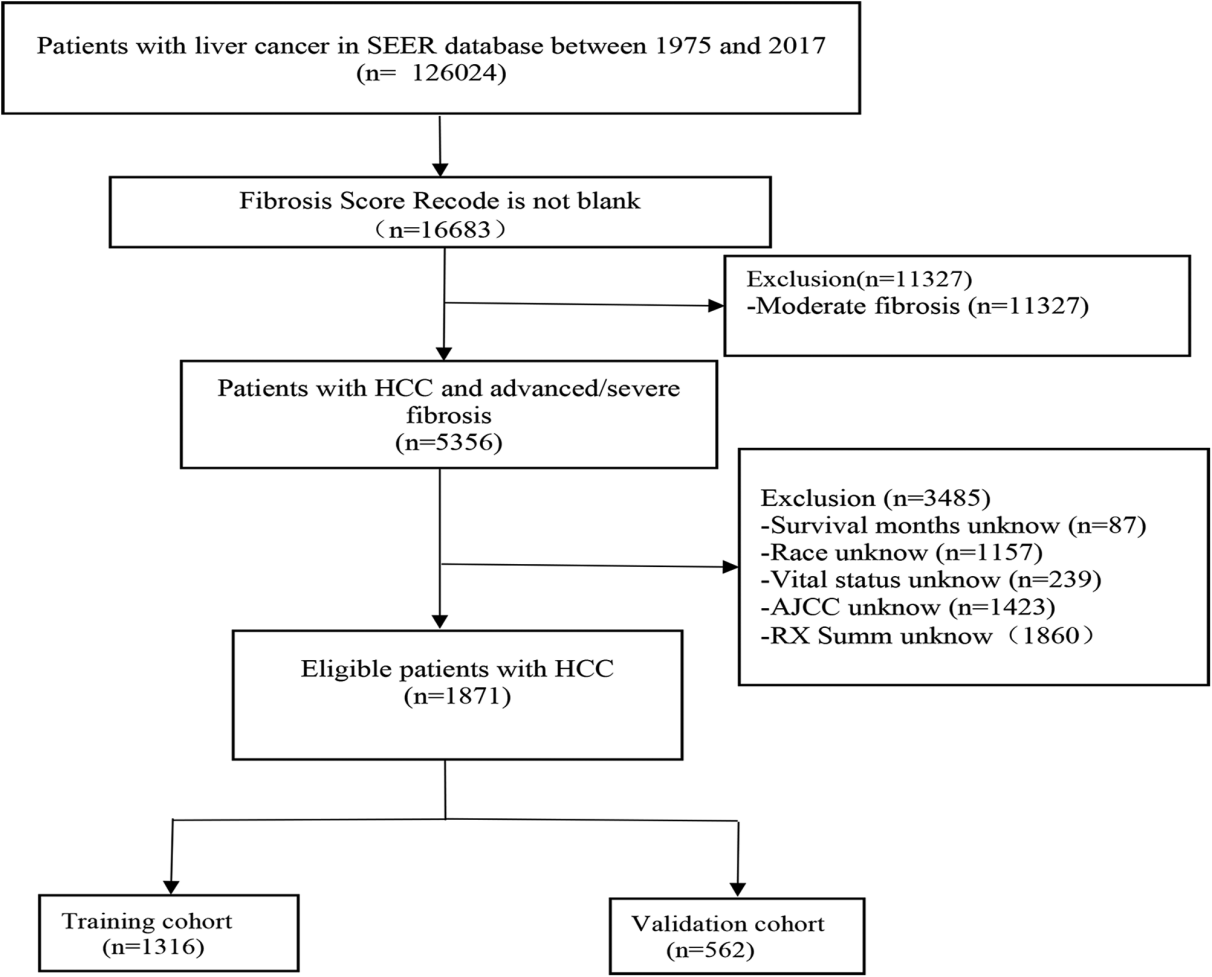
Flow diagram of the hepatocellular carcinoma patients with severe liver fibrosis with training and validation cohorts.

A total of 1,878 HCC patients with severe liver fibrosis were included in our study, with 1,316 (70%) in the training cohort and 562 (30%) in the validation cohort. The flow diagram is shown in Figure 1. Of all patients eligible for the study, there were 1,465 (77.82%) male patients and 413 female patients. There were 1,331 white and 243 black patients, which accounted for 71.14% and 12.99%, respectively. Of all the patients, 805 were treated conservatively and 300 were treated locally. A total of 394 patients underwent resection of liver masses and 379 underwent liver transplantation. A total of 1,539 patients were well-differentiated, while 339 were poorly differentiated. The baseline information related to the training and validation groups is provided in Table 1. There was no statistically significant difference between the two cohorts.

**Table 1:** Demographics and clinical characteristics of hepatocellular carcinoma patients with severe liver fibrosis at diagnosis.

Variable	Whole population		Training cohort		Validation cohort		*P* value
	*N*	%	*n*	%	*n*	%	
	1,871		1,316		562		
Age year							
<65	1,268	67.77	895	68.01	373	66.37	0.56
>65	610	32.60	421	31.99	189	33.63	
Race							
Black	243	12.99	171	12.99	72	12.81	0.16
White	1,331	71.14	945	71.81	386	68.68	
Other	304	16.25	200	15.20	104	18.51	
Sex							
F	413	22.07	290	22.04	123	21.89	0.78
M	1,456	77.82	1,026	77.96	439	78.11	
Grade							
Grade I and II	1,539	82.26	1,078	81.91	461	82.03	0.64
Grade III and IV	339	18.12	238	18.09	101	17.97	
AJCC stage^a^							
I	820	43.82	585	44.45	235	41.81	0.13
II	600	32.06	409	31.07	191	33.98	
III	301	16.08	217	16.48	84	14.94	
IV	45	2.40	105	7.97	52	9.25	
Size cm							
0–5	1,284	68.63	896	68.09	388	69.04	0.35
>5	594	31.75	420	31.91	174	30.96	
Number							
1	1,519	81.19	1,063	80.78	456	81.14	0.27
>1	359	19.19	253	19.22	106	18.86	
Extension							
Yes	375	20.04	249	18.92	436	77.58	0.61
No	1,503	80.33	1,067	81.08	126	22.42
AFP							
Positive	1,286	68.73	899	68.31	387	68.86	
Negative	592	31.64	417	31.69	175	31.14	
Surgery							
No	805	43.03	580	44.07	225	40.04	0.22
Local treatment	300	16.03	210	15.96	90	16.01	
Hepatectomy	394	21.06	263	19.98	131	23.31	
Transplant	379	20.26	263	19.98	116	20.64	
Income^b^							
Low	1,028	54.94	700	53.19	328	58.36	0.35
High	850	45.43	616	46.81	234	41.64	

^a^ *AJCC Stages: The seventh edition American Joint Committee on Cancer (AJCC) TNM staging system.*

^b^ *Income: Low, annual income <6,499$, High, annual income* ≥*6,499$.*

### Univariate and Multivariate COX Regression Analyses

Univariate COX regression analysis showed that age, race, pathological grade, AJCC stages, tumor size, AFP, surgery, tumor size, and income were all statistically significant on prognosis (*P *< 0.05) (Table 2). Multivariate analysis suggested that age, pathological grade, AJCC stages, AFP, surgery, and tumor size were independent prognostic factors affecting the CSS of HCC patients with severe liver fibrosis, which were, therefore, included in the nomogram model.

**Table 2:** The results of univariate and multivariate Cox regression analyses on variables for the prediction of CSS.

Character	Univariate		*P* Value	Multivariate		*P* Value
	HR	95%CI		HR	95%CI	
Age year						
<65	Reference			Reference		
>65	1.43	1.23–1.66	<0.001	1.24	1.06–1.45	<0.05
Race						
Black	Reference			Reference		
White	0.835	0.68–1.02	0.79	1.06	0.86–1.31	0.53
Other	0.67	0.51–0.88	0.003	0.88	0.66–1.17	0.38
Sex						
F	Reference			Reference		
M	1.19	1.00–1.43	0.04	1.03	0.86–1.24	0.68
Grade						
Grade I and II	Reference			Reference		
Grade III and IV	2.01	1.70–2.38	<0.001	1.57	1.31–1.87	<0.05
AJCC stage^a^						
I	Reference			Reference		
II	1.26	1.06–1.19	<0.001	1.41	1.17–1.70	<0.05
III	3.81	3.18–4.56	<0.001	1.46	1.15–1.85	<0.05
IV	5.02	3.37–7.47	<0.001	1.42	0.89–2.26	<0.05
Size cm						
0–5	Reference			Reference		
>5	1.28	1.14–1.54		1.21	1.17–1.46	<0.05
Number						
1	Reference			Reference		
>1	0.91	0.76–1.09	0.33	0.90	0.73–0.99	0.30
Extension						
No	Reference			Reference		
Yes	2.21	1.88–2.61	<0.001	1.28	1.05–1.58	<0.05
AFP						
Negative	Reference			Reference		
Positive	1.6	1.36–1.88	<0.001	1.19	1.01–1.41	<0.05
Surgery						
No	Reference			Reference		
Local treatment	0.35	0.29–0.44	<0.001	0.52	0.42–0.65	<0.05
Hepatectomy	0.33	0.27–0.40	<0.001	0.38	0.31–0.46	<0.05
Transplant	0.07	0.05–0.11	<0.001	0.11	0.07–0.14	<0.05
Income^b^						
Low	Reference			Reference		
High	0.8	0.69–0.92	0.03	0.85	0.73–0.99	<0.05

^a^ *AJCC Stages: The seventh edition American Joint Committee on Cancer (AJCC) TNM staging system.*

^b^ *Income: Low, annual income <6,499$, High, annual income* ≥*6,499$.*

### Construction and Validation of the Nomogram

**Figure 2:**
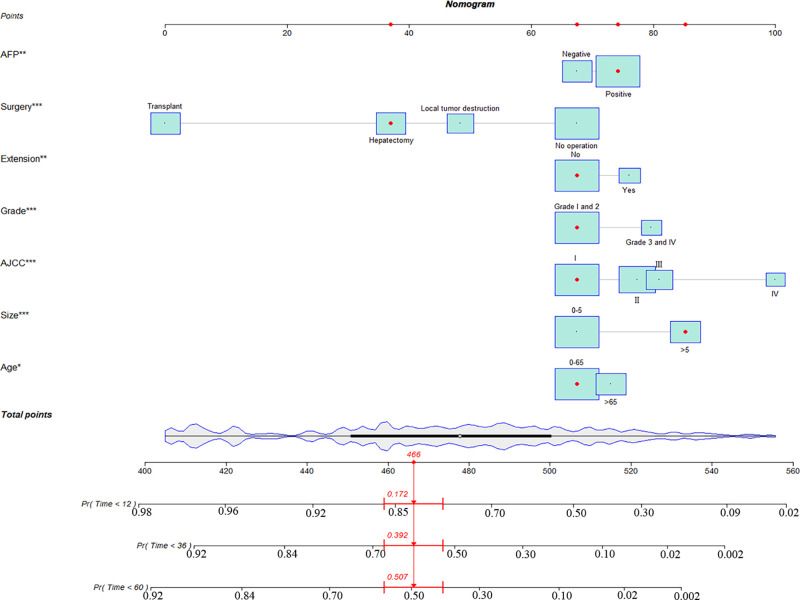
A nomogram for hepatocellular carcinoma patients with severe liver fibrosis.

**Figure 3:**
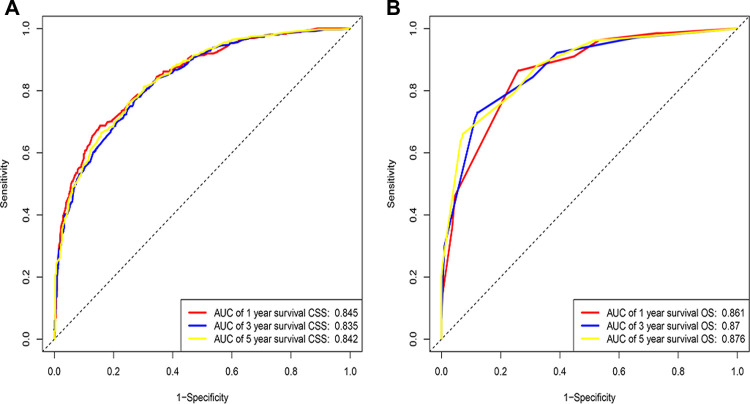
ROC of the nomogram for 1-, 3-, and 5-year prediction. (**A**) Training cohorts based on the nomogram; (**B**) Validation cohorts based on the nomogram.

**Figure 4:**
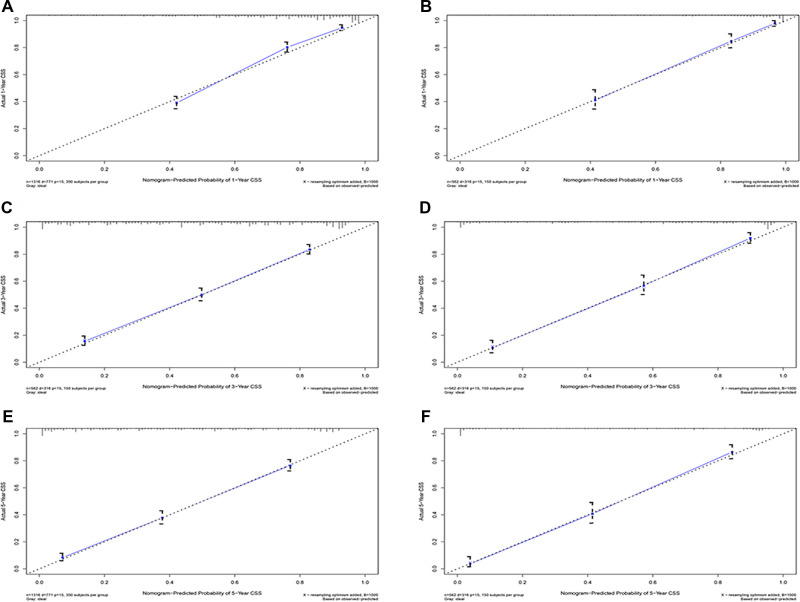
Calibration plots of 1-year, 3-year, and 5-year CSS for hepatocellular carcinoma patients with severe liver fibrosis. (**A,C,E**) Calibration plot of 1-year, 3-year, and 5-year CSS in the training cohort; (**B,C,F**) Calibration plot of 1-year, 3-year, and 5-year CSS in the training cohort; CSS, cancer-specific survival.

**Figure 5:**
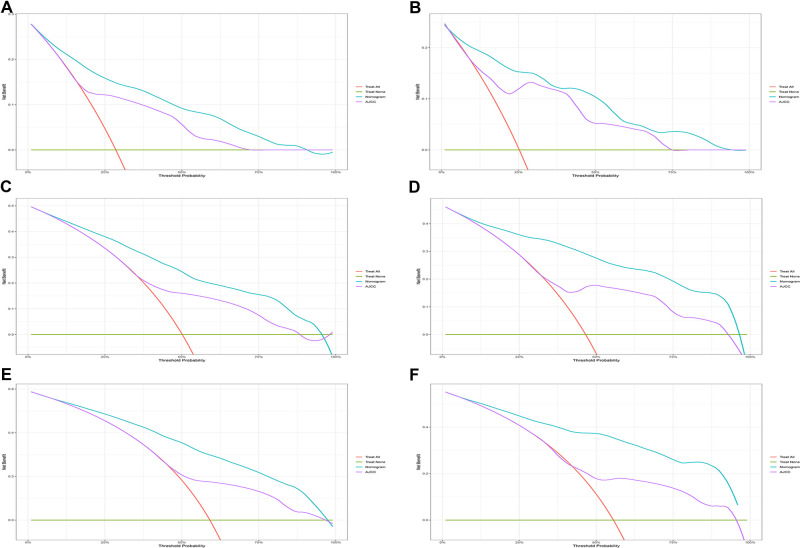
Decision curve analysis. (**A,C,E**) DCA curve of 1-year, 3-year, and 5-year CSS in the training cohort; (**B,D,F**) DCA curve of 1-year, 3-year, and 5-year CSS in the validation cohort. DCA, decision curve analysis; CSS, cancer-specific survival.

Eventually, 7 variables (age, pathological grade, AJCC stages, tumor size, AFP, surgery, and income) were selected to construct the nomogram to predict the probability of CSS in HCC patients with severe liver fibrosis (Figure 2). First, risk scores for each variable were derived based on the information of patients. The total risk score of the patient is obtained by adding the scores of all variables, and the corresponding position of the risk score of the patient can be found in the line of total scores. Finally, the probability of 1-, 3-, and 5-year CSS for HCC patients with severe liver fibrosis could be referred by drawing a straight line on the last 3 rows. The C-indexes for the training and validation cohorts were 0.781 (95% CI: 0.767–0.793) and 0.793 (95% CI: 0.779–0.798) (*P *< 0.05), respectively. The calibration curve, ROC curve, and DCA curve are shown in Figures 3–5. The 1-, 3-, and 5-year time-dependent AUCs for the training cohort were 0.845, 0.835, and 0.842, respectively, while those for the validation cohort were 0.861, 0.870, and 0.876, respectively, manifesting that the model had excellent predictive performance. In addition, the nomogram-related DCA curves at 1, 3, and 5 years in the training and validation cohorts also exhibited promising potential for clinical application and better positive net benefits. The calibration curves revealed good consistency in the probability of 1-, 3-, and 5-year CSS between the nomogram prediction and the observed results in both cohorts.

### Comparison of the Clinical Value of the Nomogram and AJCC Criteria

**Figure 6:**
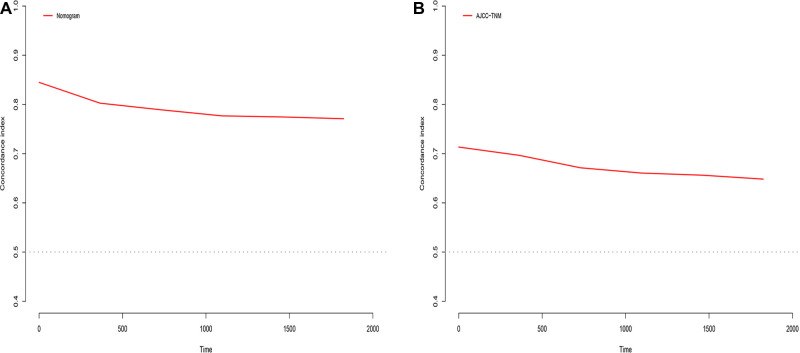
C-index analysis. (**A**) The nomogram related C-index; (**B**) AJCC staging criteria related C-index.

In the training cohort, the C-index of the nomogram was higher than that of the AJCC stage system (Figure 6). The NRIs for the 1-year, 3-year, and 5-year CSS were 0.42 (95% CI = 0.27–0.56), 0.61 (95% CI = 0.50–0.77) and 0.67 (95% CI = 0.49–0.80), respectively, in the training cohort, and 0.26 (95% CI = 0.17–0.46), 0.52 (95% CI = 0.22–0.76), and 0.72 (95% CI = 0.44–0.92), respectively, in the validation cohort. IDI (training cohort: 1-, 3-, and 5-year CSS: 0.16, 0.20, and 0.22; validation cohort: 1-, 3-, and 5-year CSS: 0.17, 0.26, and 0.30) indicated that the established nomogram significantly outperformed the AJCC staging system (*P *< 0.05) (Table 3). These results indicated that the nomogram was more accurate than predictions based on AJCC staging criteria. In addition, we compared the net benefit of the nomogram with the AJCC staging criteria. DCA curves in both the training and the validation cohorts showed that the nomogram better predicted 1-, 3-, and 5-year CSS because it added more net benefit compared with the AJCC staging criteria as well as the treat-all-patients scheme and the treat-none scheme.

**Table 3:** NRI and IDI of the nomogram and AJCC staging criteria alone in CSS prediction for hepatocellular carcinoma patients with severe liver fibrosis.

Index	Training cohort		*P* Value	Validation cohort		*P* Value
	Estimate	95%CI		Estimate	95%CI	
**NRI**						
For 1-year CSS	0.42	0.27–0.56		0.26	0.17–0.46	
For 3-year CSS	0.61	0.50–0.77		0.52	0.22–0.76	
For 5-year CSS	0.67	0.49–0.80		0.72	0.44–0.92	
**IDI**						
For 1-year CSS	0.16	0.12–0.19	<0.001	0.17	0.11–0.22	<0.001
For 3-year CSS	0.20	0.17–0.23	<0.001	0.26	0.21–0.32	<0.001
For 5-year CSS	0.22	0.19–0.28	<0.001	0.30	0.23–0.36	<0.001

**Figure 7:**
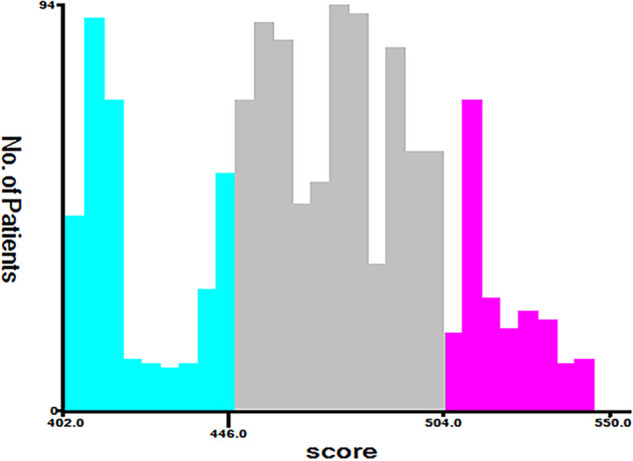
Cut-off point for risk stratification selected using X-tile.

**Figure 8:**
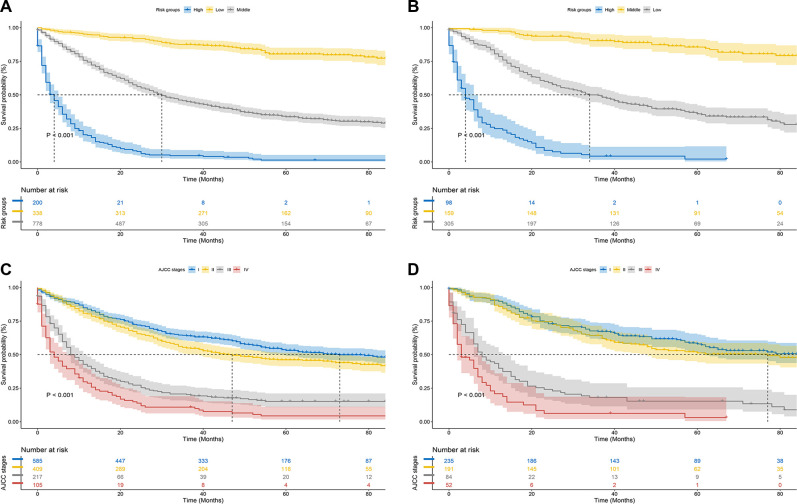
Kaplan–Meier CSS curves of hepatocellular carcinoma patients with severe liver fibrosis based on different criteria. (**A,B**) Kaplan–Meier CSS curves of training and validation cohorts based on the new risk stratification system; (**C,D**) Kaplan–Meier CSS curves of training and validation cohorts based on AJCC staging criteria.

Along with the generation of the nomogram, a risk stratification system, which was distinguished according to the calculation of the total score, was developed. All patients were classified into three risk groups: low risk (total score <446), middle risk (446 ≤ total score <504), and high risk (total score ≥504) (Figure 7). Kaplan–Meier curves presented clearly marked survival differences among patients in different risk groups. In contrast, the AJCC staging criteria model had limited ability to identify I–II and III–IV in both the training and the validation cohorts (Figure 8).

## Discussion

HCC is the sixth most common malignant cancer in incidence worldwide (17, 18), with 80%–90% of patients suffering from liver damage, chronic inflammation, and fibrous repair (19). Findings have shown that fibroblasts secreting cytokines and growth hormones can implicitly or explicitly accelerate the value-added and invasion of HCC, while a proportion of tumor-associated fibroblasts is a part of the malignant microenvironment (20, 21). HCC, combined with severe liver fibrosis, has made clinicians face substantial challenges in therapy. Meanwhile, clinical evidence for the prognosis of HCC patients with serious liver fibrosis is scarce, and there is still a shortage of risk models. Consequently, this research developed and validated a nomogram to predict the prognostic value by analyzing the demographic and clinical characteristics of HCC patients with serious liver fibrosis in the SEER database. Multiple validation results indicated that the nomogram had favorable discriminatory ability. Based on the nomogram, we developed a novel risk stratification system where patients were classified into low-risk, middle-risk, and high-risk groups. Compared with the AJCC criteria, this risk stratification system not only accurately predicted the prognosis of patients, but also provided individualized management and treatment for HCC patients with serious liver fibrosis.

Age was an independent predictor for CSS in HCC patients with serious liver fibrosis, which indicated that older age was associated with poor prognosis. Multiple studies have shown that AJCC TNM stage is an independent influencing factor for HCC, which is generally consistent with our findings (22). Patients with a higher pathological grading have a longer CSS than those with a lower pathological grading, implying that pathological grading reflects the prognosis of HCC. AFP is one of the most relevant physiological markers for screening, clinical diagnosis, effectiveness evaluation, and post-treatment monitoring in high-risk populations of liver cancer. According to current studies (23), prediction models including serum AFP can enhance the predicting recurrence of tumor after liver transplantation. Consequently, some surgeons will choose AFP models to select HCC patients who may not match Milan transplantation criteria (24). In addition, researchers are working on constructing a predictive model including AFP for HCC that involves Child B liver function. CSS is noticeably shorter in AFP-positive patients than in AFP-negative patients, which reveals that AFP exhibits a substantial predictive value in predicting long-term CSS in HCC patients with severe liver fibrosis (25, 26).

Currently, HCC is treated with chemotherapy, local therapy, mass resection, and liver transplantation. Nevertheless, there is no universally accepted treatment for HCC with severe liver fibrosis. HCC patients with severe hepatic fibrosis and those with slight liver fibrosis had diverse prognoses in previous decades (27, 28). Furthermore, it is widely considered that surgery may worsen the prognosis of HCC patients with severe liver fibrosis. Several guidelines have recommended chemotherapy, combined with local therapy, as the first-line treatment for HCC patients with severe liver fibrosis (29, 30). However, with the implementation and advancement of minimally invasive techniques in clinical practice, the rates of postoperative liver failure, mortality, and infection have decreased significantly (31, 32). Thus, surgery may provide better long-term benefits than local therapy based on acceptable short-term postoperative mortality and infection rates. According to existing studies, the five-year postoperative survival rate for HCC patients with severe liver fibrosis is up to 35% (32–34). Several of the most influential hepatobiliary institutions have argued that HCC in the presence of significant liver fibrosis is not an absolute contraindication to surgery and that patients with grade B or even C liver cancer can benefit from surgery (35, 36).

Despite the promising application of the nomogram in predicting CSS in HCC with severe liver fibrosis, this study had several limitations. First, the data lacked information on patient etiology, for instance, hepatitis B or C virus infection and alcoholic liver disease, which might affect tumor characteristics. Moreover, data on hematological indicators and surgical margins were not recorded. Finally, our model lacked a multicenter clinical sample for further validation to provide more convincing evidence.

## Conclusion

In conclusion, a practical and reliable nomogram for predicting CSS for HCC patients with severe liver fibrosis was constructed based on the significant risk factors identified in the analysis, which could effectively solve the survival paradox caused by the AJCC staging system and might help physicians make appropriate clinical decisions.

## Data Availability

The original contributions presented in the study are included in the article/Supplementary Material, and further inquiries can be directed to the corresponding author/s.
